# Mild maternal hyperglycemia in *INS*^C93S^ transgenic pigs causes impaired glucose tolerance and metabolic alterations in neonatal offspring

**DOI:** 10.1242/dmm.039156

**Published:** 2019-08-12

**Authors:** Simone Renner, Ana Sofia Martins, Elisabeth Streckel, Christina Braun-Reichhart, Mattias Backman, Cornelia Prehn, Nikolai Klymiuk, Andrea Bähr, Andreas Blutke, Christina Landbrecht-Schessl, Annegret Wünsch, Barbara Kessler, Mayuko Kurome, Arne Hinrichs, Sietse-Jan Koopmans, Stefan Krebs, Elisabeth Kemter, Birgit Rathkolb, Hiroshi Nagashima, Helmut Blum, Mathias Ritzmann, Rüdiger Wanke, Bernhard Aigner, Jerzy Adamski, Martin Hrabě de Angelis, Eckhard Wolf

**Affiliations:** 1 Chair for Molecular Animal Breeding and Biotechnology, Gene Center, LMU Munich, 81377 Munich, Germany; 2 German Center for Diabetes Research (DZD), 85764 Neuherberg, Germany; 3 MWM Biomodels, 84184 Tiefenbach, Germany; 4 Laboratory for Functional Genome Analysis (LAFUGA), Gene Center, LMU Munich, 81377 Munich, Germany; 5 Research Unit Molecular Endocrinology and Metabolism, Helmholtz Zentrum München, 85764 Neuherberg, Germany; 6 Research Unit Analytical Pathology, Helmholtz Zentrum München, 85764 Neuherberg, Germany; 7 Wageningen UR Livestock Research, de Elst 1 and CARUS Animal Facilities, Wageningen University, 6708 WD Wageningen, The Netherlands; 8 German Mouse Clinic (GMC), Helmholtz Zentrum München, 85764 Neuherberg, Germany; 9 Meiji University International Institute for Bio-Resource Research, Kawasaki 214-8571, Japan; 10 Clinic for Swine, Center for Clinical Veterinary Medicine, LMU Munich, 85764 Oberschleißheim, Germany; 11 Institute of Veterinary Pathology, Center for Clinical Veterinary Medicine, LMU Munich, 80539 Munich, Germany; 12 Department of Biochemistry, Yong Loo Lin School of Medicine, National University of Singapore, 117596 Singapore; 13 Chair of Experimental Genetics, School of Life Science Weihenstephan, Technische Universität München, 85764 Neuherberg, Germany; 14 Institute of Experimental Genetics, Helmholtz Zentrum München, 85764 Neuherberg, Germany

**Keywords:** Maternal diabetes, Pig, Transgenic, Developmental programming, Metabolomics

## Abstract

Alongside the obesity epidemic, the prevalence of maternal diabetes is rising worldwide, and adverse effects on fetal development and metabolic disturbances in the offspring's later life have been described. To clarify whether metabolic programming effects are due to mild maternal hyperglycemia without confounding obesity, we investigated wild-type offspring of *INS*^C93S^ transgenic pigs, which are a novel genetically modified large-animal model expressing mutant insulin (INS) C93S in pancreatic β-cells. This mutation results in impaired glucose tolerance, mild fasting hyperglycemia and insulin resistance during late pregnancy. Compared with offspring from wild-type sows, piglets from hyperglycemic mothers showed impaired glucose tolerance and insulin resistance (homeostatic model assessment of insulin resistance: +3-fold in males; +4.4-fold in females) prior to colostrum uptake. Targeted metabolomics in the fasting and insulin-stimulated state revealed distinct alterations in the plasma metabolic profile of piglets from hyperglycemic mothers. They showed increased levels of acylcarnitines, gluconeogenic precursors such as alanine, phospholipids (in particular lyso-phosphatidylcholines) and α-aminoadipic acid, a potential biomarker for type 2 diabetes. These observations indicate that mild gestational hyperglycemia can cause impaired glucose tolerance, insulin resistance and associated metabolic alterations in neonatal offspring of a large-animal model born at a developmental maturation status comparable to human babies.

## Abstract (precis)

**Editor's choice:** Mild maternal hyperglycemia causes impaired glucose tolerance and metabolic alterations in wild-type neonatal offspring of *INS*^C93S^ transgenic pigs, a novel large animal model for mutant *INS* gene-induced diabetes of youth (MIDY).

## INTRODUCTION

The prevalence of maternal diabetes, in particular of gestational diabetes mellitus (GDM), is rapidly increasing worldwide, primarily due to the increased prevalence of obesity. To date, the mean global prevalence of GDM in women aged 20-49 years is estimated at 16.9% (Guariguata et al., 2014) and more than 50% of pregnant women are overweight or obese (Friedman, 2018). The physiological insulin resistance (IR) in the second half of pregnancy is exacerbated in obese women and can further impair a pre-existing hyperglycemic condition or lead to GDM if insulin secretion cannot meet the increased demand (Kautzky-Willer et al., 1997).

Maternal diabetes is associated with an increased risk of adverse outcomes for mother and offspring (Wendland et al., 2012). In addition to clinical/epidemiological studies, diet-induced obese nonhuman primate (NHP) models have been used to dissect the underlying mechanisms (reviewed in Friedman, 2018). However, since these NHP models represent the entire spectrum of the metabolic syndrome it is difficult to differentiate consequences of hyperglycemia from those of obesity (Thompson et al., 2017).

Mouse models are widely used for studies of developmental programming, but their pups are born at a relatively immature stage compared with humans. Programming effects of maternal diabetes in the second half of pregnancy are therefore difficult to model in rodents. In contrast, fetal maturation in pigs is similar to that of humans (reviewed in Litten-Brown et al., 2010). To study the developmental consequences of gestational hyperglycemia, we generated *INS*^C93S^ transgenic pigs expressing mutant insulin (INS) C93S in pancreatic beta cells as a new model for mutant *INS* gene-induced diabetes of youth (MIDY) (Liu et al., 2015) and characterized their glucose homeostasis during pregnancy. The mutant insulin C93S corresponds to the Munich *Ins2*^C95S^ mutant mouse model (Herbach et al., 2007) and is similar to the human *INS*^C95Y^ mutation, which leads to permanent neonatal diabetes (Colombo et al., 2008; Stoy et al., 2010). The progression of MIDY is attributed to a so-called gain of toxic function (Colombo et al., 2008). In all three models, disruption of the A6-A11 disulfide bond within the A-chain of the insulin leads to misfolding of insulin, entrapment of insulin in the endoplasmic reticulum (ER), which also affects endogenous insulin processing and exit from the ER, leading to ER stress, and finally, β-cell death (Cunningham et al., 2017; Hodish et al., 2010). *INS*^C93S^ transgenic pigs revealed impaired glucose tolerance (IGT), reduced insulin secretion and mild fasting hyperglycemia. During pregnancy, they developed IR, but maintained a mild hyperglycemic state. Nevertheless, profound changes of carbohydrate and lipid metabolism were observed in their neonatal offspring.

## RESULTS

### Generation of *INS*^C93S^ transgenic pigs

**Fig. 1.**
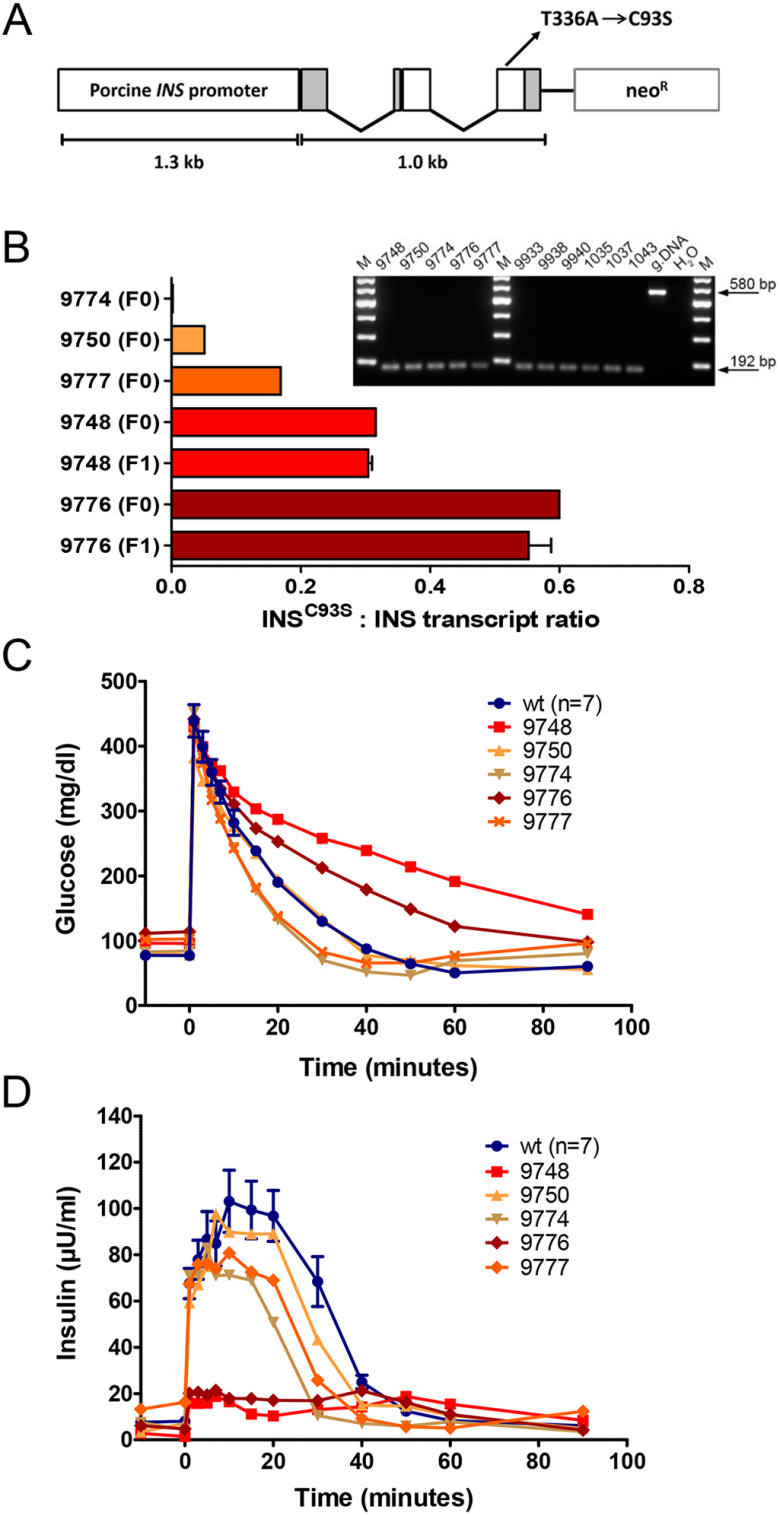
**Generation of *INS*^C93S^ transgenic pigs.** (A) Expression vector consisting of a 1.0 kb coding region of the porcine *INS* gene sequence including three exons with a T→A transition in exon 3 resulting in a Cys→Ser amino acid exchange at position 93, its essential regulatory elements and a neomycin selection cassette (neo^R^). (B) Quantification of *INS*^C93S^ and wild-type *INS* transcripts in pancreatic tissue of *INS*^C93S^ transgenic pigs by next-generation sequencing of RT-PCR amplicons. Founders 9748 and 9776 show at least 1.9-fold higher expression of the mutant *INS*^C93S^ compared with the other three founders (F0) and similar expression to their F1 offspring (F1; *n*=3). Inset shows RT-PCR products of *INS*^C93S^/*INS* transcripts in pancreatic tissue of all *INS*^C93S^ transgenic founders (left panel) and offspring from founder 9748 (9933, 9938, 9940) and founder 9776 (1035, 1037, 1043) (right panel). M, markers; gDNA, genomic DNA. (C,D) Intravenous glucose tolerance test (IVGTT) of *INS*^C93S^ transgenic founder boars (9748, 50, 74, 76, 77) and age-matched controls at 8 months of age. (C) Glucose and (D) insulin levels. Data are means±s.e.m.

**Fig. 2.**
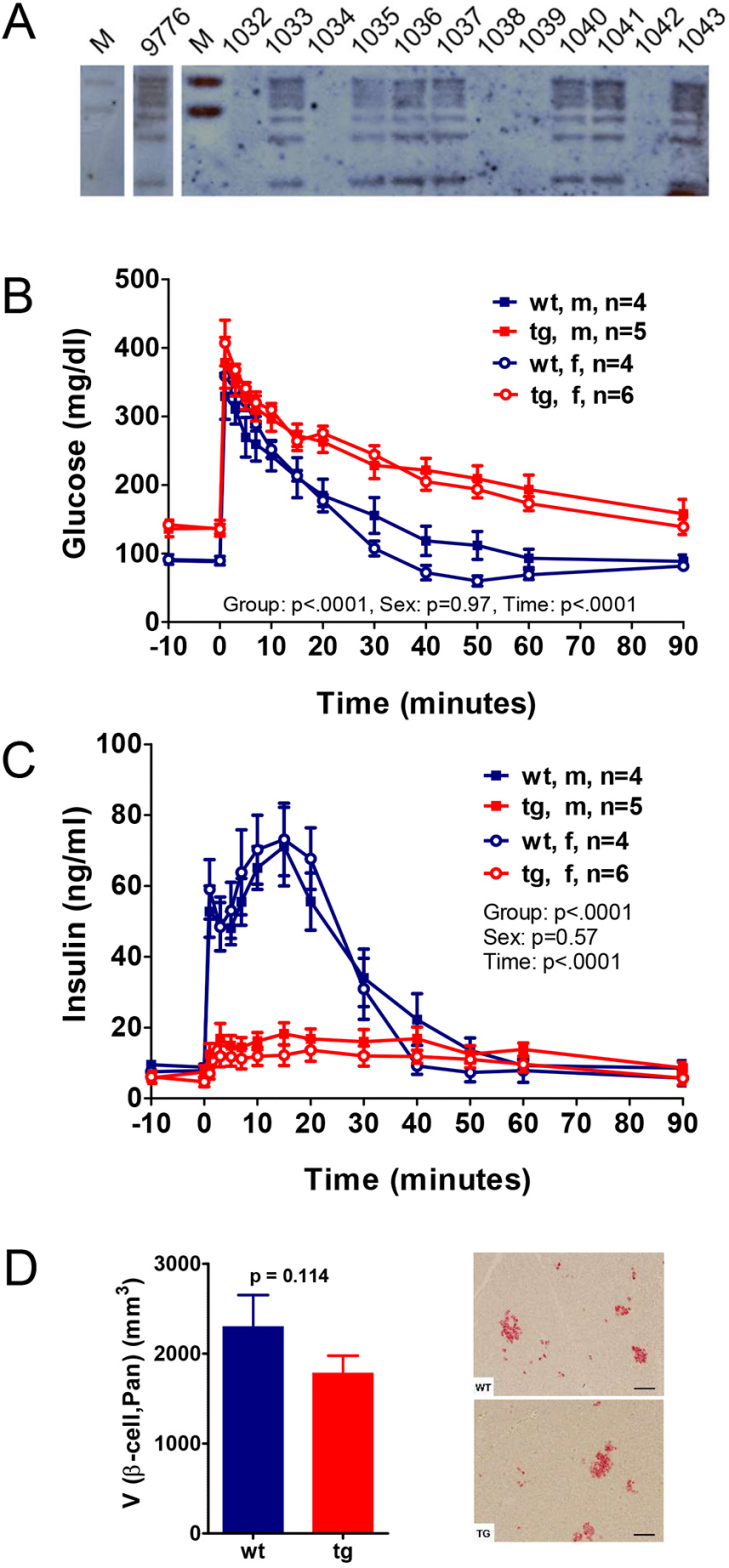
**Reduced glucose tolerance and insulin secretion in *INS*^C93S^ transgenic pigs.** (A) Southern blot analysis of PvuII-digested genomic DNA from *INS*^C93S^ transgenic pigs (1033, 1035, 1036, 1037, 1040, 1041, 1043) and littermate control animals (1032, 1034, 1038, 1039, 1042) using a [^32^P]CTP-labeled probe specific for the neomycin resistance cassette. Founder 9776 and its transgenic offspring show the same pattern, demonstrating a single integration site. (B,C) Intravenous glucose tolerance test (IVGTT) of offspring (male and female as effect was gender independent) from *INS*^C93S^ transgenic founder boar 9776 and non-transgenic littermates at 3-4 months of age. (B) Glucose and (C) insulin levels. Data are means±s.e.m. (D) Quantitative-stereological analyses of pancreatic tissue from *INS*^C93S^ transgenic pigs and non-transgenic littermates at 1 year of age. V (β-cell,Pan) is total β-cell volume in the pancreas. Images on the right are representative histological sections of pancreatic tissue stained with an α-insulin antibody from a control (WT) and an *INS*^C93S^ transgenic pig (TG). Scale bars: 50 µm.

In total, nine male *INS*^C93S^ transgenic founder animals were obtained (Landrace-Swabian Hall background) of which four died shortly after birth. Founder boars 9776 and 9748 and their respective F1 offspring revealed the highest *INS*^C93S^ to *INS* transcript ratios in the pancreas (Fig. 1B) along with reduced intravenous glucose tolerance (Fig. 1C) and highly reduced insulin secretion (Fig. 1D). Nevertheless, they showed normal growth and development as in all other founders (data not shown). Boars 9748 and 9776 were mated with WT sows. Southern blot analysis of DNA from F1 offspring revealed the same integration pattern as their respective sire (Fig. 2A), suggesting that there was only one transgene integration site in each line.

### Reduced glucose control in *INS*^C93S^ transgenic pigs

IVGTTs and MMGTTs in offspring of founder boars 9748 and 9776 were performed at 3-4 months of age and revealed similar reduced levels of glucose tolerance and insulin secretion (Fig. 2B,C, Figs S1 and S2) in male and female *INS*^C93S^ transgenic pigs. In addition, fasting blood glucose concentrations were significantly elevated [+56% (141.0±6.6 vs 90.2±2.6 mg/dl; *P*<0.0001) and +19% (100.8±2.6 vs 84.5±0.4 mg/dl; *P*<0.001) for offspring of 9776 and 9748 vs age-matched controls, respectively], while fasting insulin concentrations were not altered. Offspring of founder 9776 only were used for further analyses.

At 7 months of age, i.e. when pigs had reached sexual maturity, glucose control of TG pigs had further deteriorated, with significantly (*P*=0.02) decreased fasting plasma insulin (FPI) concentrations (7.0±0.6 vs 10.3±1.1 µU/ml in WT littermates; Fig. S1). Female TG pigs revealed elevated fasting glucose concentrations and showed a further reduced glucose tolerance with less insulin secretion compared with their male counterparts (Fig. S1C-F). Volume density of β-cells in the pancreas and total β-cell volume of 1-year-old TG pigs tended to be lower compared with levels in WT littermates but the difference was not significant (Fig. 2D).

### Reduced insulin sensitivity and β-cell function during late gestation

**Fig. 3.**
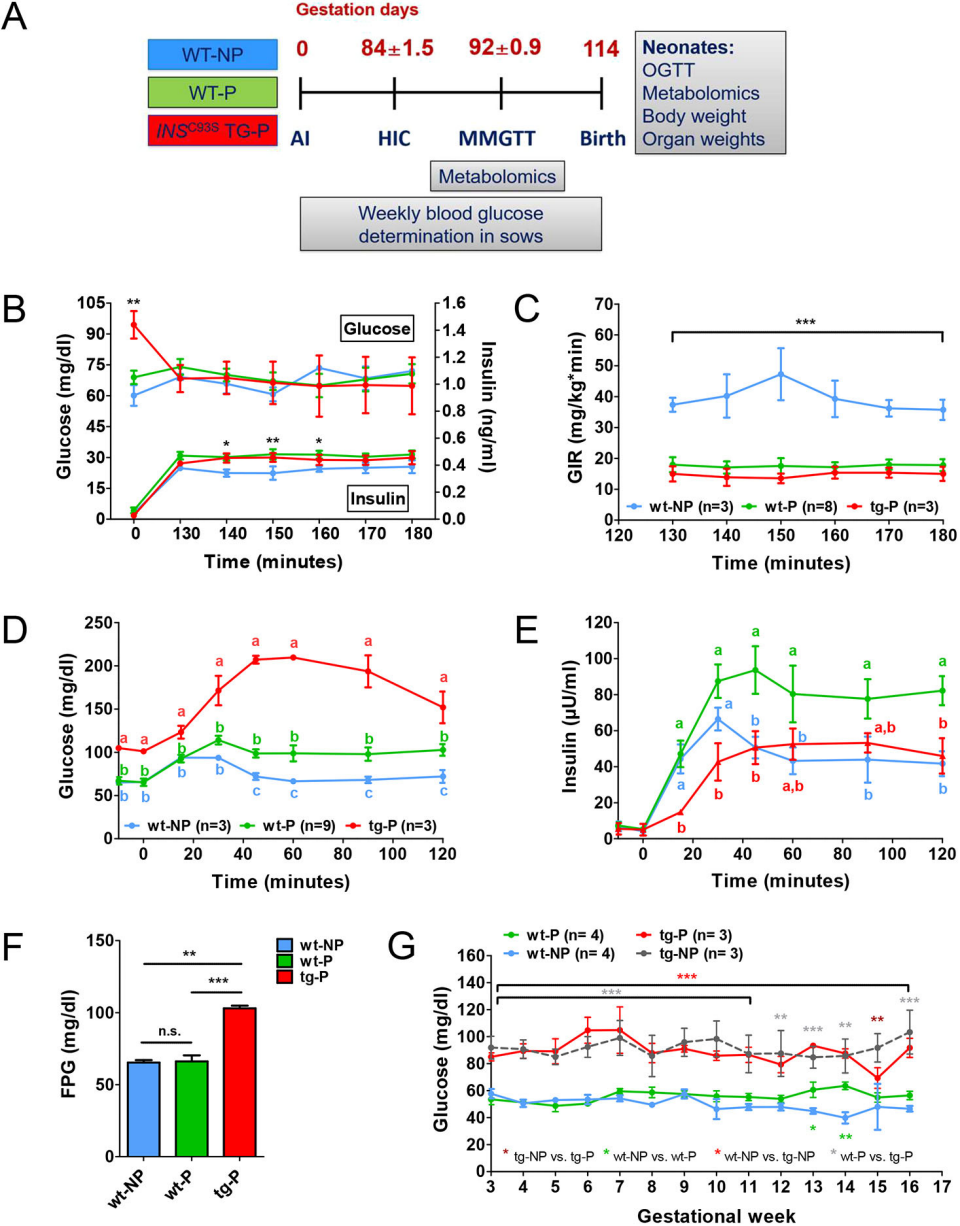
**Reduced insulin sensitivity and insufficient compensatory capacity to maintain glucose control in *INS*^C93S^ transgenic pigs during pregnancy.** (A) Study outline of the evaluation of glucose control in sows during pregnancy and in neonates after birth. WT-NP, non-pregnant control; WT-P, pregnant control; TG-P, pregnant *INS*^C93S^ transgenic; AI, artificial insemination; HIC, hyperinsulinemic-euglycemic clamp; MMGTT, mixed-meal glucose tolerance test; OGTT, oral glucose tolerance test. (B,C) HIC of pregnant *INS*^C93S^ transgenic (TG-P) and non-transgenic (WT-P) sows as well as of non-pregnant controls (WT-NP). (B) Glucose and insulin levels. (C) Glucose infusion rate (GIR). (D,E) MMGTT in TG-P, WT-P and WT-NP sows. (D) Glucose and (E) insulin concentrations. (F,G) Fasting blood glucose concentrations within the third trimester (time-point of the MMGTT) (F) and throughout pregnancy (G). Data are means±s.e.m.; *P<0.05, **P<0.01, ***P<0.001; different letters indicate significant difference between groups.

HICs and MMGTTs were performed in pregnant (P) TG (TG-P) and age-matched WT sows (WT-P) in the third trimester (Fig. 3A). Non-pregnant (NP) WT sows (WT-NP) served as controls. TG-P sows did not differ from WT-P sows in any clinical-chemical and metabolomic parameters except for significantly elevated methionine sulfoxide concentrations in the fasting and elevated glucose concentrations in the fasting and postprandial state (Tables S1, S3). During HIC, steady-state glucose concentrations (130-180 min) were not significantly different between WT-NP, WT-P and TG-P sows, but insulin concentrations between 140 and 160 min were significantly higher in WT-P compared with WT-NP sows (Fig. 3B). The glucose infusion rate (GIR) was significantly reduced in both WT-P and TG-P sows (AUC for GIR: −56% and −63% compared with WT-NP sows), indicating an insulin-resistant state (Fig. 3C). During MMGTT, WT-P sows showed a small but significant impairment of glucose tolerance associated with a distinctly increased insulin secretion (AUC glucose: +29%; AUC insulin: +62% compared with WT-NP sows; Fig. 3D,E). In TG-P sows, glucose tolerance was severely impaired (AUC glucose: +129%; AUC insulin: −3% compared with WT-NP sows; Fig. 3D,E). Fasting plasma glucose (FPG) was significantly elevated in TG-P sows, but unaltered in WT-P compared with WT-NP sows (Fig. 3F). During the entire course of pregnancy, FPG of TG-P sows did not increase significantly compared with levels in non-pregnant TG sows (Fig. 3G).

### Impaired glucose tolerance and metabolic alterations in neonatal offspring of hyperglycemic sows

OGTTs were performed in neonatal piglets of normoglycemic WT sows (NG piglets) and hyperglycemic TG sows (HG piglets) prior to the first colostrum uptake. The latter group included the wild-type, but not the transgenic offspring. Plasma samples from time points 0 min and 120 min relative to glucose load (representing the fasting and insulin-stimulated state) were analyzed by clinical chemistry and targeted metabolomics.

**Fig. 4.**
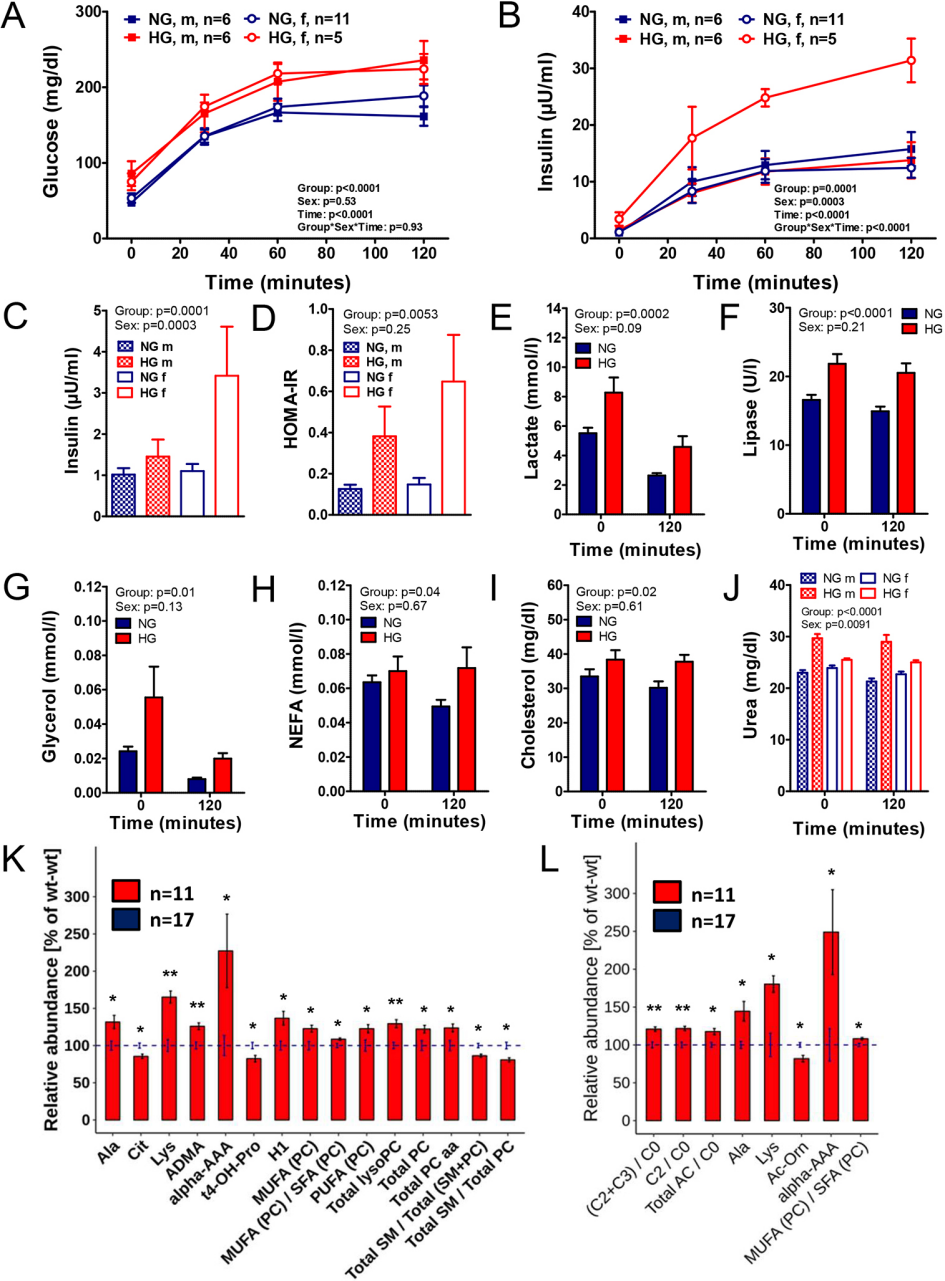
**Reduced glucose tolerance and increased insulin secretion in neonatal piglets born to *INS*^C93S^ transgenic sows.** (A,B) Oral glucose tolerance test in piglets prior to first colostrum uptake born to normoglycemic wild-type sows (NG) or to hyperglycemic *INS*^C93S^ transgenic sows (HG). (A) Glucose, (B) insulin, (C) fasting insulin, (D) homeostatic model assessment of insulin resistance (HOMA-IR). (E-J) Clinical chemical parameters in NG and HG piglets in the fasting (0 min) and insulin-stimulated (120 min relative to an oral glucose load) state. (E) Lactate, (F) lipase, (G) glycerol, (H) NEFA, (I) cholesterol and (J) urea concentrations. (K,L) Significantly different abundance of metabolites in plasma samples of NG compared with HG piglets in the (K) fasting state and (L) insulin-stimulated (120 min relative to an oral glucose load) state. (K,L) Significantly different metabolites and metabolic indicators are shown as a percentage of the NG mean (blue striped line). The s.d. for each metabolite and genotype is indicated with error bars. *P<0.05, **P<0.01.

Compared with NG piglets, male and female HG piglets revealed a significantly impaired glucose tolerance to a similar extent (Fig. 4A), associated with increased insulin secretion (Fig. 4B) and fasting hyperinsulinemia (Fig. 4C) in female but not male HG piglets. Homeostatic model assessment of insulin resistance (HOMA-IR) was increased 3-fold in male and 4.4-fold in female HG piglets compared with their male and female NG counterparts (Fig. 4D). Plasma lactate, lipase, glycerol, NEFA, cholesterol, urea and bilirubin-D concentrations were significantly elevated in HG piglets (Fig. 4E-J, Table S4). A sex effect was observed for urea levels, with a pronounced elevation in male HG piglets (Fig. 4J).

Targeted metabolomics of fasting plasma samples revealed significantly increased concentrations of hexoses and glycerophospholipids [lyso-phosphatidylcholines and total phosphatidylcholines (PC), with mainly diacylglycerols (PC aa), and a higher proportion of mono- and polyunsaturated PCs] in samples from HG compared with NG piglets. The ratio of total sphingomyelins (SM) to total PC was significantly reduced. Additionally, HG piglets exhibited changes in their amino acid metabolism characterized by a distinct elevation in lysine (160.5 vs 97.2 µM, *P*=0.002) along with increased concentrations of α-aminoadipic acid (α-AAA). Furthermore, plasma concentrations of alanine and asymmetric dimethylarginine (ADMA) were increased, while those of citrulline and trans-4-hydroxyproline (t4-OH-Pro) were reduced in HG versus NG piglets (Fig. 4K).

After the oral glucose bolus, the ratio of total and short-chain acylcarnitines (C2+C3) to carnitine was significantly increased in HG piglets. Total lyso-phosphatidylcholines and the concentrations of lysine, alanine and α-AAA were significantly increased, while those of tyrosine and acetyl-ornithine (Ac-Orn) were decreased in HG compared with NG piglets (Fig. 4L). The complete metabolomic data set is presented in Table S5. Body and organ weights of neonatal HG and NG piglets were not significantly different (Table S6).

## DISCUSSION

*INS*^C93S^ transgenic pigs represent a novel large animal model for MIDY (Liu et al., 2015). The C93S mutation was chosen on the basis of the analogous Munich *Ins2*^C95S^ mutant mouse model (Herbach et al., 2007) and the human *INS*^C95Y^ mutation (Colombo et al., 2008; Stoy et al., 2010), which lead to an early diabetic phenotype, i.e. permanent neonatal diabetes. The phenotype of *INS*^C93S^ transgenic pigs was less severe than that in a line of *INS*^C94Y^ transgenic pigs (Renner et al., 2013), most probably due to a lower level of mutant *INS* transgene expression (mutant *INS*:WT *INS* transcript ratio 55% and 78% in *INS*^C93S^ and *INS*^C94Y^ transgenic pigs, respectively).

To establish a genetically modified large animal model for maternal diabetes, we initially characterized the metabolic status of *INS*^C93S^ transgenic pigs during late gestation. A genetically modified model is superior to pre-existing surgically and chemically induced large-animal models in this field, as it is less invasive, has no direct impact on the exocrine pancreas and a minor phenotypic inter-individual variance (Renner et al., 2016).

Pigs are of translational value for the evaluation of metabolic programming effects as they are very similar to humans when considering their nutrition, digestive and pancreas physiology (Renner et al., 2016), as well as their propensity for the development of obesity and the metabolic syndrome (Renner et al., 2018). Genetic engineering in combination with dietary intervention allows the evaluation of metabolic programming effects due to maternal diabetes, obesity or a combination of both. Also, evaluation of glucose homeostasis by e.g. glucose tolerance tests and more sophisticated tests such as glucose clamps can be evaluated in the unrestrained and unstressed animal via long-term catheterization techniques and animal training. Despite differences in pig versus human placenta (epithelial-chorial vs hemochorial), transfer of relevant nutrients such as glucose, amino acids and at least partially of fatty acids takes place in both species (Litten-Brown et al., 2010). Decisive phases of early embryonic development are also similar, as major genome activation takes place between the 4-cell and 8-cell stage in pig and human compared with the 2-cell stage in mouse (Simmet et al., 2018). Also, in rodents, late phases of fetal development are generally not exposed to an altered maternal metabolism as their pups are born at a more immature state compared with pigs and humans (Litten-Brown et al., 2010).

The mild maternal hyperglycemia as present in *INS*^C93S^ transgenic sows closely represents the human situation where glucose concentrations of diabetic mothers are very tightly regulated as a result of current recommendations from professional societies (American Diabetes Association, 2018). During late pregnancy, insulin sensitivity of WT and TG sows was reduced by 50%, a similar level to that observed in pregnant women (Catalano et al., 1992b). In the MMGTT, insulin secretion in TG-P sows raised to the level of WT-NP sows, but did not meet the elevated insulin demand.

Male and female newborns of mildly hyperglycemic sows exhibited IGT, whereas only females showed fasting hyperinsulinemia and increased insulin secretion. HOMA-IR was increased in male and female piglets but to a greater extent in females. Sex differences in metabolic programming have been reported in rodents, non-human primates and humans (Dearden et al., 2018). In accordance with the pig model described here, maternal diabetes was associated with pronounced glucose intolerance, hyperinsulinemia and elevated HOMA-IR in rodent (Samuelsson et al., 2013; Vickers et al., 2011) and human (Krishnaveni et al., 2010) females compared with male offspring. Female human offspring revealed an increased sensitivity to even mildly elevated maternal glucose levels compared with levels in males (Regnault et al., 2013).

Mild maternal hyperglycemia, even in the absence of maternal and neonatal increased fat mass/obesity, is sufficient for metabolic disturbances in the offspring. In humans, the development of fetal IR *in utero* was associated with increased fetal fat mass and had a strong correlation with maternal pre-gravid BMI and degree of IR (Catalano et al., 2009).

Early neonatal metabolism in humans is characterized by a drop in insulin as a result of the adrenergic stimulation associated with delivery, leading to increased lipolytic activity and a switch from maternal glucose supply to absolute dependence on active gluconeogenesis, because glucose supply from milk covers only 40-50% of the glucose utilization rate (Girard, 1989). Increased availability of typical gluconeogenic precursors such as lactate, alanine, glycerol and NEFAs provided by increased lipase activity in HG piglets might be explained by the reduced insulin sensitivity allowing higher rates of gluconeogenesis. Furthermore, increased lactate concentrations in HG piglets might be caused by an earlier switch to energy supply from anaerobic glycolysis as a result of fetal IR and increased metabolic rates (Taricco et al., 2009). Elevated plasma urea concentration in HG piglets contrast with human studies showing no effect of maternal diabetes on urea synthesis (Kalhan, 1993).

The most pronounced metabolic changes of HG piglets were increased concentrations of lysine (+65%) and α-aminoadipic acid (2-fold). α-Aminoadipic acid is a product of lysine metabolism/breakdown (Requena et al., 2001) and is a potential biomarker for type 2 diabetes (Wang et al., 2013). Its application was associated with increased insulin secretion in cells and rodents (Wang et al. 2013), serving as a potential compensatory mechanism for early IR.

HG piglets also revealed profound differences in their lipid metabolism. Similarly to HG piglets, increased lyso-PC concentrations were reported in fetuses from lean mothers with GDM (Lu et al., 2018) as well as a positive correlation to maternal glycemia in obese mother–offspring pairs (Patel et al., 2018). In contrast, decreased lyso-PC concentrations were associated with obesity, IR (Lehmann et al., 2013) and diabetes (Barber et al., 2012) in humans.

In addition to differences present in the fasting state, HG piglets revealed an increased ratio of total and short chain (C2+C3) acylcarnitines to free carnitine, indicative of an increased import of fatty acids into mitochondria and increased β-oxidation rate. Increased plasma acylcarnitine concentrations have been observed in association with IR in humans (Schooneman et al., 2013). Enhanced β-oxidation in an insulin-stimulated state normally dominated by glucose oxidation can occur due to lack of insulin's inhibiting effect on fat oxidation in the IR state as accumulation of acetyl-CoA from β-oxidation inhibits pyruvate dehydrogenase, glycolysis, and ultimately, glucose uptake into the cell (Kelley and Mandarino, 2000). Moreover, increased abundance of C2 might be part of a compensatory mechanism restoring glucose uptake into the cell by enabling export of acetyl-CoA as membrane-permeable C2. Increased concentrations of C2 are contraindicative of acylcarnitine deacylation in the kidney (Guder and Wagner, 1990).

Mild maternal hyperglycemia did not alter birth weight in HG piglets. In humans, maternal obesity and increased neonatal fat mass have emerged as major determinants of variation in neonatal body weight. Maternal BMI is strongly associated with fetal overgrowth and increased fat mass independent of glycemia in the HAPO study (Catalano et al., 2012). Owing to a comparatively high body fat content of 16%, differences in fat mass can account for ∼50% of variations in human birth weight (Catalano et al., 1992a). Unlike humans, rodents and pigs have a lower body fat content of 1% and 2%, making them more sensitive for hypoglycemia-associated starvation shortly after birth, but possibly less sensitive for the development of increased birth weight in the absence of obesity.

Mild maternal hyperglycemia in normal-weight *INS*^C93S^ transgenic pigs leads to IGT, IR and profound associated metabolic alterations in their non-transgenic offspring. Therefore, *INS*^C93S^ transgenic pigs represent a valuable model for the dissection of developmental programming originating from maternal hyperglycemia, obesity and their cumulative effects in a highly standardized manner.

## MATERIALS AND METHODS

### Generation of *INS*^C93S^ transgenic pigs

All experiments were performed according to the German Animal Welfare Act with permission from the responsible authority (Government of Upper Bavaria), following the ARRIVE guidelines and Directive 2010/63/EU. The mutant insulin *INS*^C93S^ expression vector consists of a 1.0 kb coding region of the porcine *INS* gene sequence, including three exons with a T→A transition in exon 3 resulting in a Cys→Ser amino acid exchange at position 93, its essential regulatory elements and a neomycin selection cassette (*neo*) (Fig. 1A). Pools of stable transfected cell clones were used for somatic cell nuclear transfer (SCNT). SCNT and embryo transfer were performed as previously described (Kurome et al., 2015). Genotyping was carried out by PCR using the transgene-specific primers 5′-TGATTCCCACTTTGTGGTTC-3′ and 5′-GTGGATGTGGAATGTGTGC-3′, and Southern blot analysis using PvuII-digested genomic DNA and a [^32^P]CTP-labeled probe specific for the neomycin resistance cassette (Fig. 2A). For expression analysis of the *INS*^C93S^ transgene, total RNA was extracted from pancreatic tissue (RNeasy^®^ total RNA isolation Kit; Qiagen), digested with DNaseI (Roche) and reverse-transcribed with SuperScript^TM^ II Reverse Transcriptase (Invitrogen) using random hexamer primers. *INS*^C93S^ and wild-type *INS* cDNAs were amplified using the primers 5′-CGGGAGGCGGAGAACCCTCA-3′ and 5′-CCCTCAGGGGCGGCCTAGTT-3′ and the ratio of the products was determined by next-generation sequencing (Genome Analyzer IIx; Illumina; ≥10,000 reads per sample).

### Animals and study design during the pregnant state

Animals (Landrace-Swabian-Hall background) were housed under controlled conditions, fed a commercial pig diet once daily with free access to water. The study outline is shown in Fig. 3A. Briefly, female primiparous transgenic (TG) and wild-type (WT) littermate sows were estrus synchronized as described previously (Kurome et al., 2015) and artificially inseminated with semen from the same boar. WT non-pregnant sows served as controls. Within the third trimester, hyperinsulinemic-euglycemic clamps (HICs) and mixed meal glucose tolerance tests (MMGTTs) were performed. Blood glucose concentrations of sows were determined on a weekly basis during pregnancy. Birth was induced by a single injection of a prostaglandin-F_2α_-agonist (Estrumate^®^, MSD). At the day of birth, oral glucose tolerance tests (OGTTs) were performed in newborn piglets prior to the first colostrum uptake. Piglets were euthanized within 24 h of birth for organ sampling (see below) and determination of absolute and relative organ weight.

### Blood parameters

Blood samples for clinical chemical analyses were taken after an overnight fasting period of 18-20 h and at time point 120 min during an MMGTT from sows within the third trimester (gestation day 92±0.9) and from piglets on the day of birth prior to first colostrum uptake, as well as at time point 120 min during an OGTT. Blood was collected into EDTA-coated tubes and processed as previously described (Renner et al., 2010). Plasma insulin concentrations were determined using a species-specific RIA (Merck Millipore) or ELISA (Mercodia). Clinical chemical parameters (Table S1 and S4) were determined from EDTA-plasma using an AU480 autoanalyzer (Beckman-Coulter) and adapted reagent kits from Beckman-Coulter, Randox or Wako Chemicals. Blood glucose of sows during pregnancy was determined weekly using a Precision Xceed^®^ glucometer (Abbott).

### Intravenous, oral and mixed meal glucose tolerance tests

Intravenous glucose tolerance tests (IVGTTs) and MMGTTs were performed as previously described (Renner et al., 2010). Briefly, animals were fitted with central venous catheters prior to the tests. For the IVGTT a 0.5 g/kg body weight (BW) bolus of 50% glucose solution and for the MMGTT 2 g/kg BW glucose and 150-400 g of a commercial pig diet dependent on the BW of the respective age group was administered. In neonatal pigs, OGTTs were performed prior to first colostrum uptake. Therefore, a bolus of 50% glucose solution (2 g/kg BW) was administered via a gastric tube. Blood samples were taken at indicated time points relative to the glucose load from the catheter (sows) or directly from the jugular vein (piglets).

### Hyperinsulinemic-euglycemic clamp

For stress-free, frequent blood sampling in unrestrained animals, animals were fitted with central venous (glucose and insulin infusion) and arterial (blood sampling) catheters. After an 18-h fasting period, insulin (Insuman^®^ rapid) was infused as a prime dose (0.5 mU/kg BW) for 2.5 min followed by a constant infusion rate of 1 mU/kg BW/min (Koopmans et al., 2006) for a total period of 180 min. Blood glucose concentrations were clamped at 75 mg/dl. Blood glucose was determined every 5 min and glucose infusion rate adjusted accordingly. Blood samples during steady state for determination of glucose infusion rate (GIR, mg/kg×min) were collected every 10 min starting at 130 min. Calculated GIR was normalized to plasma insulin concentrations of the respective time point.

### Targeted metabolomics

The targeted metabolomics approach was based on LC-ESI-MS/MS and FIA-ESI-MS/MS measurements by AbsoluteIDQ™ p180 kit (BIOCRATES Life Sciences AG, Innsbruck, Austria). The assay allows simultaneous quantification of 188 metabolites out of 10 µl plasma, and includes free carnitine, 39 acylcarnitines (Cx:y), 21 amino acids (19 proteinogenic+citrulline+ornithine), 21 biogenic amines, hexoses (sum of hexoses; about 90-95% glucose), 90 glycerophospholipids, 14 lysophosphatidylcholines (lysoPC), 76 phosphatidylcholines (PC), and 15 sphingolipids (SMx:y) (Table S2). The abbreviations Cx:y are used to describe the total number of carbons and double bonds of all chains, respectively (for more details, see Römisch-Margl et al., 2012). The method of AbsoluteIDQ™ p180 kit has been shown to conform with the Food and Drug Administration's Guidance for Industry – Bioanalytical Method Validation (May 2018) (US Department of Health and Human Services et al., 2018), which implies proof of reproducibility within a given error range. Measurements were performed as described by the manufacturer in manual UM-P180. Analytical specifications for limits of detection (LOD) and evaluated quantification ranges, further LOD for semi-quantitative measurements, identities of quantitative and semi-quantitative metabolites, specificity, potential interferences, linearity, precision and accuracy, reproducibility and stability were described in the Biocrates manual AS-P180. The LODs were set to three times the values of the zero samples (PBS). The lower and upper limits of quantification were determined experimentally by Biocrates. The assay procedures of the AbsoluteIDQ^TM^ p180 kit have been described in detail previously (Römisch-Margl et al., 2012; Zukunft et al., 2013). Metabolite nomenclature is provided in Table S2. Sample handling was performed by a Hamilton Microlab STARTM robot (Hamilton Bonaduz AG, Bonaduz, Switzerland) and an Ultravap nitrogen evaporator (Porvair Sciences, Leatherhead, UK) alongside standard laboratory equipment. Mass spectrometric analyses were done on an API 4000 triple quadrupole system (Sciex Deutschland GmbH, Darmstadt, Germany) equipped with a 1200 Series HPLC (Agilent Technologies Deutschland GmbH, Böblingen, Germany) and a HTC PAL auto sampler (CTC Analytics, Zwingen, Switzerland) controlled by the software Analyst v.1.6.1. Data evaluation for quantification of metabolite concentrations and quality assessment was performed with the MetIDQ™ software package, which is an integral part of the AbsoluteIDQ™ kit. Internal standards serve as reference for the calculation of metabolite concentrations (µM).

### Necropsy, pancreas sampling, immunohistochemistry and quantitative stereology

Pigs were anesthetized by injection of ketamine (Ursotamin^®^, Serumwerke Bernburg) and azaperone (Stresnil^®^, Elanco) and euthanized under anesthesia by intravenous injection of T61^®^ (Intervet) and immediately subjected to necropsy. The pancreas was explanted *in toto*, weighed, and samples were taken by a systematic random sampling procedure (Renner et al., 2010). Samples were routinely processed for paraffin histology. Insulin-containing cells were identified by immunohistochemistry (insulin-containing cells stained with guinea pig anti-porcine insulin antibody, dilution 1:1000, A0564 Dako Cytomation and secondary antibody goat anti-guinea pig IgG, dilution 1:100, 6090-04 Southern Biotech with Vector^®^ Red, Vector Laboratories Inc. as chromogen), and the volume density of β-cells within the pancreas (*V*_β-cell_/*V*_pancreas_) and the total β-cell volume (*V*_β-cell_) were quantified (Streckel et al., 2015). Piglets born to TG and WT sows were necropsied within 24 h of birth and selected organs, as well as the carcass, were weighed.

### Statistics

All data are presented as means±s.e.m. unless otherwise indicated. The results of glucose tolerance tests (IVGTT, MMGTT, OGTT) and glucose infusion rate (GIR) during the hyperinsulinemic-euglycemic clamp, as well as blood glucose concentrations during pregnancy, were statistically evaluated by ANOVA (Linear mixed models; SAS 8.2; PROC MIXED), taking into account the fixed effects of Group [normoglycemic (NG) vs hyperglycemic (HG) for neonates, WT vs TG for all others], the Status (non-pregnant vs pregnant) for sows, Sex (male vs female) if applicable, Time (relative to glucose application or glucose/insulin infusion) and the interaction Group×Time or Group×Status×Time or Group×Sex×Time as well as the random effect of Animal. Body weight, absolute and relative organ weights as well as clinical chemical parameters were statistically evaluated by ANOVA (General linear models; SAS 8.2) taking into account the fixed effects of Group, Sex, if applicable, Time, and the interaction Group×Time, Group×Sex and Group×Sex×Time. Area under the curve (AUC) for insulin/glucose was calculated using GraphPad Prism^®^ software (v.5.02). AUCs and all data from quantitative-stereological analyses were evaluated by Mann-Whitney *U*-test using GraphPad Prism^®^ software. *P*-values less than 0.05 were considered significant. For metabolomics data, missing values in the metabolomics measurements were imputed using half the value of the minimum measurement for that metabolite. Metabolites with more than 50% missing values were excluded. Normalization and batch effect removal was performed by calculating a plate factor using the means of individual metabolites per plate and multiplying each metabolite with the corresponding value. Student's two-tailed *t*-tests were used on log-transformed and Pareto-scaled (centered and divided by square root of standard deviation) metabolites for calculating significance. For multiple testing correction, the Benjamini-Hochberg procedure was employed and a false discovery rate (FDR) below 0.05 was considered significant.

## Supplementary Material

Supplementary information
