# Supplementary material for: Mild maternal hyperglycemia in INSC93S transgenic pigs causes impaired glucose tolerance and metabolic alterations in neonatal offspring
Source: Dis Model Mech. 2019 Aug 12;12(8):dmm039156. doi: 10.1242/dmm.039156 (PMC6737953; doi:10.1242/dmm.039156)
Supplement: Supplementary information [file dmm-12-039156-s1.pdf]

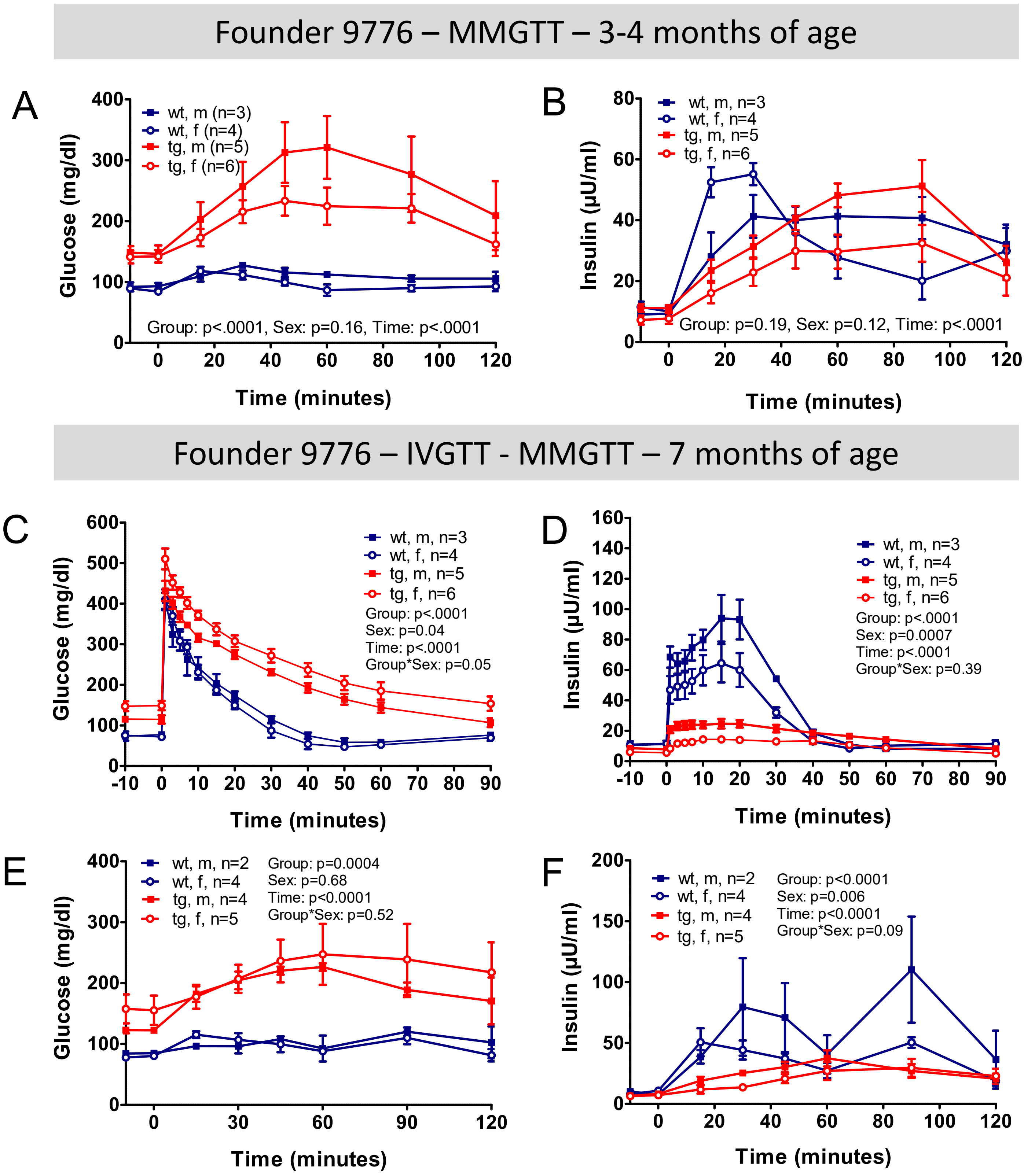

Figure S1: MMGTT from male and female offspring of founder boar 9776 at three to four months of age and IVGTT and MMGTT at seven months of age. Data are means ± SEM.

Disease Models & Mechanisms • Supplementary information

Founder 9748 – IVGTT - MMGTT – 3 months of age

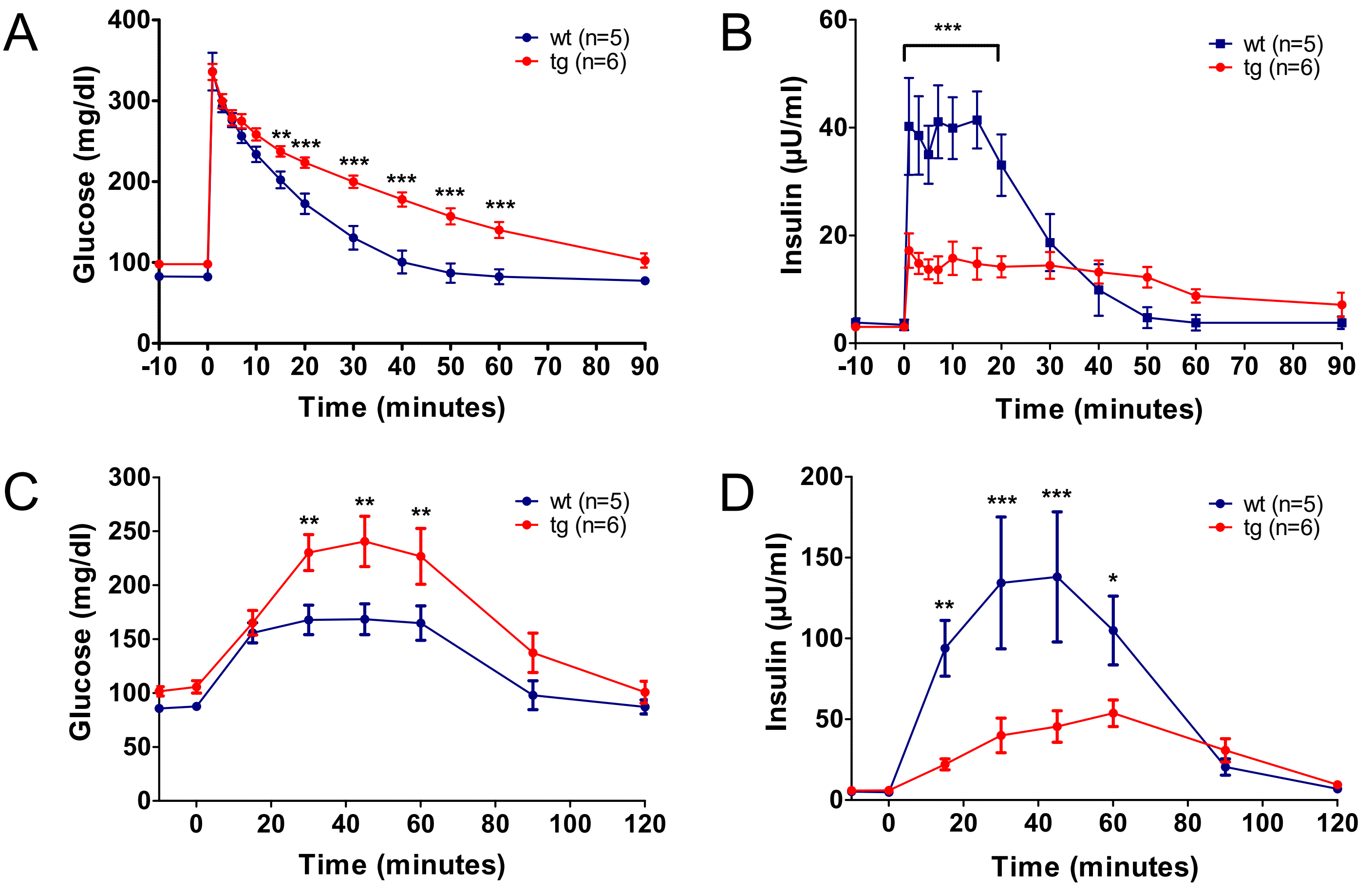

Figure S2: IVGTT and MMGTT from male and female offspring of founder boar 9748 at three months of age. Data are means ± SEM; *: $p<0.05$, **: $p<0.01$, ***: $p<0.001$.

Table S1: Clinical-chemical parameters in pregnant wild-type (WT-P) and pregnant *INS*^{C93S} transgenic (TG-P) sows. Clinical-chemical parameters in pregnant wild-type (WT-P, n=6) and pregnant *INS*^{C93S} transgenic (TG-P, n=3) sows during late gestation (gestation day 92 ± 1.1) at time-points 0 and 120 minutes of a MMGTT. Longitudinal data were evaluated by ANOVA (General Linear Models) taking the fixed effects of Group, Time and the interaction Group*Time into account.

Parameter	Time	Group		Analysis of variance		
		WT-P	TG-P	Group	Time	Group x Time
**Glucose** [mg/dl]	**0**<br>**120**	71.3 ± 5.7<br>111.7 ± 7.6	109.5 ± 3.1<br>154.0 ± 15.2	0.0004	0.0002	0.82
**Lactate** [mmol/l]	**0**<br>**120**	0.89 ± 0.05<br>1.9 ± 0.2	0.87 ± 0.06<br>1.9 ± 0.4	0.94	0.0002	0.99
**LDH** [U/l]	**0**<br>**120**	333.8 ± 14.1<br>359.2 ± 11.4	520.3 ± 176.7<br>485.6 ± 141.4	0.06	0.95	0.70
**Bicarbonate** [mmol/l]	**0**<br>**120**	20.2 ± 0.5<br>21.2 ± 0.7	21.2 ± 1.1<br>20.4 ± 0.3	0.90	0.89	0.21
**Cholesterol** [mg/dl]	**0**<br>**120**	67.0 ± 4.2<br>64.3 ± 4.5	74.5 ± 4.8<br>70.5 ± 4.0	0.19	0.52	0.90
**LDL** [mg/dl]	**0**<br>**120**	43.3 ± 2.7<br>42.4 ± 3.1	49.4 ± 3.5<br>45.7 ± 3.4	0.19	0.51	0.68
**HDL** [mg/dl]	**0**<br>**120**	25.7 ± 1.9<br>25.2 ± 2.0	28.0 ± 1.82<br>25.5 ± 1.3	0.54	0.48	0.66
**Triglycerides** [mg/dl]	**0**<br>**120**	28.2 ± 2.9<br>22.1 ± 2.9	27.9 ± 2.7<br>27.2 ± 2.4	0.47	0.31	0.42
**Glycerol** [mmol/l]	**0**<br>**120**	0.05 ± 0.03<br>0.004 ± 0.0003	0.02 ± 0.006<br>0.004 ± 0.0009	0.4	0.16	0.39
**NEFA** [mmol/l]	**0**<br>**120**	0.6 ± 0.3<br>0.04 ± 0.005	0.3 ± 0.06<br>0.04 ± 0	0.49	0.12	0.49
**ASAT** [U/l]	**0**<br>**120**	18.8 ± 1.8<br>18.3 ± 1.6	26.3 ± 9.9<br>24.3 ± 7.5	0.15	0.78	0.87
**ALAT** [U/l]	**0**<br>**120**	34.0 ± 3.2<br>33.3 ± 3.4	41.3 ± 5.8<br>39.3 ± 5.0	0.04	0.65	0.82
**GGT** [U/l]	**0**<br>**120**	33.3 ± 1.5<br>36.7 ± 2.9	31.7 ± 8.3<br>28.0 ± 5.7	0.22	0.97	0.40
**Bilirubin** [mg/dl]	**0**<br>**120**	0.2 ± 0.02<br>0.1 ± 0.003	0.1 ± 0.01<br>0.1 ± 0	0.64	0.26	0.88
**Lipase** [U/l]	**0**<br>**120**	2.1 ± 0.2<br>2.4 ± 0.2	4.2 ± 1.0<br>2.8 ± 1.1	0.03	0.32	0.12
**Albumin** [g/dl]	**0**<br>**120**	3.8 ± 0.07<br>3.8 ± 0.04	3.9 ± 0.04<br>3.7 ± 0.1	0.67	0.12	0.16
**Total protein** [g/dl]	**0**<br>**120**	6.7 ± 0.1<br>6.8 ± 0.08	7.0 ± 0.1<br>6.6 ± 0.08	0.49	0.05	0.14
**Creatinine** [mg/dl]	**0**<br>**120**	1.6 ± 0.04<br>1.6 ± 0.14	1.7 ± 0.06<br>1.6 ± 0.08	0.42	0.94	0.43
**Urea** [mg/dl]	**0**<br>**120**	23.3 ± 1.9<br>23.6 ± 2.0	28.3 ± 2.3<br>27.4 ± 1.1	0.07	0.88	0.79
**CRP** [mg/l]	**0**<br>**120**	11.2 ± 1.7<br>11.1 ± 1.5	12.0 ± 7.8<br>12.6 ± 8.9	0.79	0.95	0.94

Disease Models & Mechanisms • Supplementary information

Table S2: List of metabolites measured with the AbsoluteIDQ® p180 Kit GAC, Helmholtz Zentrum München

Click here to Download Table S2

Table S3: ***A.*** Targeted metabolomics findings in pregnant wild-type **(WT-P, n=9)** and *INS*^{C93S} transgenic **(TG-P, n=3)** sows in the fasting condition. ***B.*** Targeted metabolomics findings in pregnant wild-type **(WT-P, n=9)** and *INS*^{C93S} transgenic **(TG-P, n=3)** sows in the insulin-stimulated condition (120 minutes time-point of the mixed-meal glucose tolerance test, MMGTT).

Click here to Download Table S3

Table S4: Clinical-chemical parameters in piglets born to wild-type (NG) and *INS*^{C93S} transgenic sows (HG). Clinical-chemical parameters in wild-type piglets born to wild-type sows (NG, n=17) and wild-type piglets born to *INS*^{C93S} transgenic sows (HG, n=11) at the day of birth prior to and 120 minutes following an oral glucose load (2g / kg body weight). Longitudinal data were evaluated by ANOVA (General Linear Models; SAS 8.2) taking the fixed effects of Group, Sex, Time, and the interaction Group*Sex and Group*Sex*Time into account.

Parameter	Time	Group		Analysis of variance				
		wt-wt	wt-tg	Group	Sex	Time	Group x Sex	Group x Sex x Time
**Glucose** [mg/dl]	**0**	53.7 ± 4.33	81.2 ± 12.8	0.0015	0.84	<0.0001	0.15	0.72
	**120**	187.3 ± 10.8	239.0 ± 17.6					
**Lactat** [mmol/l]	**0**	5.51 ± 0.37	8.27 ± 1.03	0.0002	0.09	<0.0001	0.03	0.75
	**120**	2.64 ± 0.16	4.6 ± 0.7					
**LDH** [U/l]	**0**	312.7 ± 9.8	310.8 ± 27.0	0.43	0.16	<0.0001	0.77	0.49
	**120**	430.6 ± 18.9	447.8 ± 25.8					
**Cholesterol** [mg/dl]	**0**	33.5 ± 2.1	38.4 ± 2.7	0.02	0.61	0.45	0.56	0.77
	**120**	30.2 ± 1.9	37.8 ± 2.0					
**LDL** [mg/dl]	**0**	25.8 1.41	28.3 3.3	0.14	0.65	0.53	0.31	0.96
	**120**	23.6 1.37	28.0 2.1					
**HDL** [mg/dl]	**0**	12.7 ± 0.9	17.2 ± 1.3	0.001	0.07	0.19	0.33	0.95
	**120**	11.7 ± 0.8	15.5 ± 0.9					
**Triglycerides** [mg/dl]	**0**	11.7 ± 0.53	14.4 ± 1.7	0.11	0.24	0.0002	0.07	0.32
	**120**	9.36 ± 0.42	9.8 ± 0.5					
**Glycerol** [mmol/l]	**0**	0.02 ± 0.003	0.06 ± 0.02	0.01	0.13	0.002	0.11	0.34
	**120**	0.008 ± 0.0009	0.02 ± 0.003					
**NEFA** [mmol/l]	**0**	0.06 ± 0.004	0.07 ± 0.008	0.04	0.67	0.32	0.91	0.48
	**120**	0.05 ± 0.004	0.07 ± 0.01					
**ASAT** [U/l]	**0**	13.6 ± 0.94	15.8 ± 1.2	0.56	0.27	0.0003	0.67	0.71
	**120**	21.8 ± 2.07	22.4 ± 2.2					
**GGT** [U/l]	**0**	89.9 ± 3.0	92.6 ± 2.5	0.28	0.2	0.02	0.25	0.78
	**120**	78.2 ± 2.7	86.0 ± 3.8					
**Bilirubin** [mg/dl]	**0**	0.06 ± 0.005	0.06 ± 0.005	0.05	0.17	0.06	0.36	0.75
	**120**	0.07 ± 0.008	0.1 ± 0.01					
**Bilirubin-D** [mg/dl]	**0**	0.017 0.006	0.02 0.008	0.01	0.05	0.02	0.41	0.73
	**120**	0.025 0.003	0.04 0.02					
**Lipase** [U/l]	**0**	16.6 ± 0.7	21.8 ± 1.4	<0.0001	0.21	0.27	0.11	0.89
	**120**	14.9 ± 0.7	20.5 ± 1.4					
**Albumin** [g/dl]	**0**	0.75 ± 0.02	0.7 ± 0.06	0.26	0.61	0.19	0.75	0.67
	**120**	0.66 ± 0.02	0.7 ± 0.05					
**Total protein** [g/dl]	**0**	2.26 ± 0.03	2.3 ± 0.1	0.18	0.82	0.004	0.80	0.55
	**120**	1.98 ± 0.04	2.2 ± 0.08					
**Creatinine** [mg/dl]	**0**	1.08 ± 0.06	1.0 ± 0.04	0.91	0.06	0.90	0.01	0.60
	**120**	1.04 ± 0.06	1.1 ± 0.04					
**Urea** [mg/dl]	**0**	23.6 ± 0.6	28.3 ± 0.9	<0.0001	0.0091	0.06	<0.0001	0.88
	**120**	22.2 ± 0.4	27.2 ± 1.0					
**Bicarbonate** [mmol/l]	**0**	19.4 ± 1.46	21.6 ± 3.3	0.28	0.56	0.14	0.13	0.88
	**120**	21.9 ± 1.1	25.6 ± 2.6					

Disease Models & Mechanisms • Supplementary information

Table S5: ***A.*** Targeted metabolomics findings in wild-type piglets born to normoglycemic wild-type sows **(NG, n=17)** and wild-type piglets born to hyperglycemic *INS*^{C93S} transgenic sows **(HG, n=11)** in the fasting condition. ***B.*** Targeted metabolomics findings in wild-type piglets born to normoglycemic wild-type sows **(NG, n=17)** and wild-type piglets born to hyperglycemic *INS*^{C93S} transgenic sows **(HG, n=11)** in the insulin-stimulated condition (120 minutes relative to an oral glucose load).

Click here to Download Table S5

Table S6: Body and organ weights in piglets born to wild-type and *INS*^{C93S} transgenic sows. Body and (relative) organ weights in wild-type piglets born to wild-type sows **(NG)** and wild-type piglets born to *INS*^{C93S} transgenic sows **(HG)** at day one of age. Data were evaluated by ANOVA (General Linear Models; SAS 8.2) taking the fixed effect of Group into account.

**Parameter**	**LS Means**		**P value**
	**HG**	**NG**	
Body weight (g)	1473.806	1476.686	0.974
CRL (cm)	28.223	28.559	0.785
*rel*CRL (%)	2.550	2.583	0.676
Pancreas (g)	2.109	1.998	0.549
*rel*Pancreas (%)	0.141	0.138	0.649
Liver (g)	44.338	40.929	0.429
*rel*Liver (%)	3.001	2.762	0.067
Heart (g)	12.593	11.536	0.349
*rel*Heart (%)	0.851	0.789	0.066
Kidneys (g)	5.505	5.815	0.572
*rel*Kidneys (%)	0.371	0.395	0.265
Lungs (g)	43.024	46.475	0.280
*rel*Lungs (%)	2.982	3.196	0.222
Spleen (g)	2.130	2.093	0.888
*rel*Speen (%)	0.142	0.141	0.980
Carcass (g)	1149.384	1142.208	0.936
*rel*Carcass (%)	78.267	77.783	0.758

rel: relative; CRL: crown-rump length
